# Prevalence of insomnia symptoms and their associated factors in patients treated in outpatient clinics of four general hospitals in Guangzhou, China

**DOI:** 10.1186/s12888-018-1808-6

**Published:** 2018-07-18

**Authors:** Wei Zheng, Xin-Ni Luo, Hai-Yan Li, Xiao-Yin Ke, Qing Dai, Chan-Juan Zhang, Chee H. Ng, Gabor S. Ungvari, Yu-Tao Xiang, Yu-Ping Ning

**Affiliations:** 1 The Affiliated Brain Hospital of Guangzhou Medical University (Guangzhou Huiai Hospital), Guangzhou, China; 2 Department of Psychiatry, University of Melbourne, Melbourne, VIC Australia; 3 University Notre Dame Australia, Perth, Australia; 4 Division of Psychiatry, School of Medicine, University of Western Australia, Perth, Australia; 5 Unit of Psychiatry, Faculty of Health Sciences, University of Macau, Macao SAR, China

**Keywords:** Insomnia symptoms-medical outpatients-China

## Abstract

**Background:**

Data on the prevalence of insomnia symptoms in medical outpatient clinics in China are lacking. This study examined the prevalence of insomnia symptoms and their socio-demographic correlates in patients treated at medical outpatient clinics affiliated with four general hospitals in Guangzhou, a large metropolis in southern China.

**Method:**

A total of 4399 patients were consecutively invited to participate in the study. Data on insomnia and its socio-demographic correlates were collected with standardized questionnaires.

**Results:**

The prevalence of any type of insomnia symptoms was 22.1% (95% confidence interval (CI): 20.9–23.3%); the prevalence of difficulty initiating sleep was 14.3%, difficulty maintaining sleep was 16.2%, and early morning awakening was 12.4%. Only 17.5% of the patients suffering from insomnia received sleeping pills. Multiple logistic regression analysis revealed that male gender, education level, rural residence, and being unemployed or retired were negatively associated with insomnia symptoms, while lacking health insurance, older age and more severe depressive symptoms were positively associated with insomnia symptoms.

**Conclusions:**

Insomnia symptoms are common in patients attending medical outpatient clinics in Guangzhou. Increasing awareness of sleep hygiene measures, regular screening and psychosocial and pharmacological interventions for insomnia are needed in China.

**Trial registration:**

ChiCTR-INR-16008066. Registered 8 March 2016.

**Electronic supplementary material:**

The online version of this article (10.1186/s12888-018-1808-6) contains supplementary material, which is available to authorized users.

## Background

Insomnia symptoms, including difficulty initiating sleep (DIS), difficulty maintaining sleep (DMS) and early morning awakening (EMA), are major health problems worldwide [1–4]. Insomnia is associated with daytime fatigue, reduced activity, absenteeism, poor quality of life (QOL), and significant medical and societal costs [5, 6]. The prevalence of sleep problems in the general population ranges from 6 to 76.3% across various countries using different time-frame, measurements and sampling methods [7–10]. Commonly reported contributing factors to insomnia symptoms include advanced age, female gender, low personal income and educational level, and psychiatric or somatic conditions [9, 11, 12].

In order to develop preventive strategies and allocate health resources, it is important to examine the prevalence of insomnia symptoms. In Western countries the prevalence of insomnia symptoms has been extensively examined in both the general population [13] and medical conditions, such as epilepsy [14] and stroke [15]. Sociocultural and economic factors play an important role in determining the duration and quality of sleep [16, 17]. Therefore, characteristics of insomnia symptoms need to be examined in ethnically and socio-culturally diverse populations. In China, patterns of insomnia symptoms in the general population have been well-documented. A recent meta-analysis of 17 studies found that the pooled prevalence of insomnia symptoms in the Chinese general population was 15.0% [1].

Patients treated in medical outpatient clinics usually have insomnia symptoms resulting in poor sleep quality [18]. A cohort study from 1999 to 2010 found there was a significant increase in prevalence of sleep problems in hospital outpatients [19]. The prevalence of insomnia symptoms in outpatient clinics has been mixed across studies [12, 20]. For example, the prevalence of insomnia symptoms was 11.7% in Japanese (*n* = 6277) general hospital outpatients [12] while the corresponding figure was 38.5% in China [20].

Data on insomnia symptoms in patients attending medical outpatient clinics their related risk factors are lacking in China. This study aimed to determine the one-month prevalence of different types of insomnia symptoms in Chinese outpatients treated in clinics attached to general hospitals, and also explore their associations with demographic characteristics. In China, there are very few outpatient clinics located in the community; most clinically stable patients with medical conditions attend outpatient clinics attached to general hospitals.

In China, according to the size and specialization of clinical care and research, hospitals are classified into the following three levels [21]: 1. Level-I hospitals are small, community hospitals that only provide basic medical care; 2. Level-II hospitals are regional medical centres that deliver complex health care to patients with severe medical and psychiatric conditions; 3. Level-III hospitals have the highest staff-patient ratio and usually the best medical resources.

## Methods

### Study sites and participants

This was a multi-center, cross-sectional survey conducted in Guanzhou, a large metropolis in southern China, between March 15, 2016 and June 30, 2016. Patients treated in the neurological, cardiovascular, gastrointestinal, and gynecological outpatient clinics attached to four major level-III hospitals (see Additional file 1) in Guangzhou (the First Affiliated Hospital of Guangzhou University of Traditional Chinese Medicine, Guangzhou Panyu Central Hospital, Nanfang Hospital affiliated with Southern Medical University, and the Third Affiliated Hospital of Sun Yet-Sen University) were consecutively invited to participate in the study if they fulfilled the following inclusion criteria: (1) age of 18 years or above; (2) ability to communicate in Chinese (Mandarin or Cantonese) and (3) understand the purpose and content of the survey.

The study protocol was approved by the Ethics Committee of the Affiliated Brain Hospital of Guangzhou Medical University. Written informed consent was obtained from each patient.

### Assessments and evaluation

Information on basic socio-demographic characteristics was collected with a questionnaire by trained psychiatrists and PhD students in clinical psychology at the outpatient clinics of the participating hospitals. All investigators attended a training that introduced the study objectives, procedures of the data collection, and ethical issues of the survey.

Information of three basic types of insomnia symptoms occurring in the past month was ascertained by asking three standard questions, that had been used in previous studies [2, 22]: DIS: *Did you ever have difficulties in falling sleep?*; DMS: ‘*Did you ever have difficulties in maintaining sleep?*’; and EMA: ‘*Did you ever wake up in the middle of the night or early morning and have difficulties in falling asleep again?*’. There were three options to reply to these questions: ‘*No*’, ‘*Sometimes*’ and ‘*Often*’. If patients answered “often” to any of the three questions, they were classified into the “suffering from any insomnia symptoms” group.

The presence and severity of depressive symptoms were assessed with the validated Chinese version of the Patient Health Questionnaire 9 (PHQ-9) [23, 24], which has nine items; each was rated from 0 to 3. Higher scores indicated more severe depressive symptoms. The PHQ-9 score of 10 was used as a cut-off for “having depressive symptoms”, which has satisfactory sensitivity (0.77) and specificity (0.76) in Chinese medical outpatients [23]. Anxiety symptoms were evaluated with the validated Chinese version of the 7-item Generalized Anxiety Disorder (GAD-7) [25, 26]. Each item of the GAD-7 was rated from 0 to 3. Higher scores indicated more severe anxiety symptoms. Patients with a GAD-7 total score of 10 was considered as “having anxiety symptoms”; this cut-off point that has good sensitivity (86.2%) and specificity (95.5%) in Chinese population [25].

### Statistical analysis

All data were analyzed using the SPSS, Version 20.0 statistical software (SPSS Inc., Chicago, United States). The comparison between patients with and without insomnia symptoms with regard to demographic characteristics was conducted by chi-square tests, two independent samples *t*-tests or Mann-Whitney U test, as appropriate. Multiple logistic regression analysis with the “Enter” method was performed to determine the independent relationships between insomnia symptoms and socio-demographic variables. Each type of insomnia symptoms was separately entered as the dependent variable, while given the large sample size, all socio-demographic and clinical characteristics were the independent variables. The level of significance was set at 0.05 (two-tailed). As this was an exploratory study, the significance level was not corrected.

## Results

A total of 5284 patients were invited to participate in the survey of whom 4399 completed all the questionnaires yielding a participation rate of 83.3%. The prevalence of any type of insomnia symptoms was 22.1% (95% confidence interval (CI): 20.9–23.3%). The weighted prevalence of any type of insomnia symptoms according to the distribution of patients in the participating hospitals was 21.0% (95% CI: 20.3–21.6%). In the whole sample, the prevalence of DIS, DMS and EMA was 14.3, 16.2 and 12.4%, respectively. Of the 973 patients who reported insomnia symptoms, the percentage of patients with one, two, and three types of insomnia symptoms were 39.6, 27.3, and 33.1%, respectively. Only 17.5% patients (170/973) with insomnia symptoms were taking any type of “sleeping pills (sedatives and hypnotics)”.

Table 1 shows the comparison between patients with and without insomnia symptoms with respect to demographic characteristics. Patients with insomnia symptoms were more likely to be females, older in age, had lower personal income, lower education level, family history of psychiatric disorders, and more severe anxiety and depressive symptoms. Significant differences were also found between the two groups regarding marital and employment status.

**Table 1:** Demographic and clinical characteristics of the study sample

	Total sample (*n* = 4399)		Insomnia symptoms (*n* = 973)		Without insomnia symptoms (*n* = 3426)		Statistics		
	N	%	N	%	N	%	χ^2^	df	*p*
Male gender	1503	34.2	302	31.0	1201	35.1	5.4	1	**0.02**
Marital status							36.9	2	**< 0.001**
Single	728	16.5	147	15.1	581	17.0			
Married	3528	80.2	765	78.6	2763	80.6			
Divorced/widowed	143	3.3	61	6.3	82	2.4			
Rural residence	2631	59.8	559	57.5	2072	60.5	2.9	1	0.09
Employment status							16.4	2	**< 0.001**
Employed	2900	65.9	589	60.5	2311	67.5			
Unemployed	800	18.2	201	20.7	599	17.5			
Retired	699	15.9	183	18.8	516	15.1			
Living alone	415	9.4	99	10.2	316	9.2	0.8	1	0.3
Personal monthly income >/=6000 (Yuan)	951	21.6	178	18.3	773	22.6	8.1	1	**0.004**
No health insurance	2825	64.2	645	66.3	2180	63.6	2.3	1	0.1
Family history of psychiatric disorders	217	4.9	87	8.9	130	3.8	42.8	1	**< 0.001**
Current drinking	959	21.8	214	22.0	745	21.7	0.03	1	0.8
Current smoking	594	13.5	136	14.0	458	13.4	0.2	1	0.6
	Mean	SD	Mean	SD	Mean	SD	T / Z	df	*p*
Age (years)	41.8	15.9	44.4	16.1	41.0	15.8	−5.9	4397	**< 0.001**
Education (years)	10.3	4.2	9.6	4.2	10.5	4.2	5.8	4397	**< 0.001**
GAD-7 total	3.2	4.2	5.3	5.4	2.6	3.6	−15.9	---^a^	**< 0.001**
PHQ-9 total	3.6	4.3	6.5	5.5	2.8	3.5	−22.8	---^a^	**< 0.001**

^a^=Mann-Whitney U test; Bolded values are *p* < 0.05; GAD-7 = Generalized Anxiety Disorder Scale-7; PHQ-9 = Patient Health Questionnaire-9; SF-12 = Medical Outcomes Study Short Form 12

In China, the age of ≥50 years has often been used as the cutoff value for “older adults” [27, 28]. Following other studies [27–29], the prevalence figures of various types of insomnia symptoms by gender and age groups are listed in Table 2. DIS (14.9% vs. 12.9%), DMS (17.1% vs. 14.4%), and EMA (12.9% vs. 11.2%) were more frequent in females than in males. Compared to younger patients (< 50 years), older patients (>/=50 years) reported more frequent DIS (17.0% vs. 13.1%), DMS (19.8% vs. 14.7%), and EMA (17.2% vs. 10.4%).

**Table 2:** Prevalence of any type of insomnia symptoms by age and sex

Age (years) (*n* = 628)	DIS (n = 628)			Age (years) (*n* = 711)	DMS (n = 711)			Age (years) (*n* = 544)	EMA (n = 544)			Age (years) (n = 4399)	Any insomnia symptoms (n = 4399)		
	Female % (95% CI)	Male % (95% CI)	Total % (95% CI)		Female % (95% CI)	Male% (95% CI)	Total % (95% CI)		Female% (95% CI)	Male% (95% CI)	Total % (95% CI)		Female% (95% CI)	Male% (95% CI)	Total % (95% CI)
< 50 (*n* = 410)	13.4 (11.9–14.8)	12.6 (10.5–14.7)	13.1 (12.0–14.3)	< 50 (*n* = 458)	15.4 (13.9–16.9)	13.1 (11.0–15.3)	14.7 (13.4–15.9)	< 50 (*n* = 324)	10.7 (9.4–12.0)	9.6 (7.8–11.5)	10.4 (9.3–11.5)	< 50 (n = 324)	20.8 (19.1–22.5)	18.7 (16.3–21.2)	20.1 (18.7–21.5)
>/=50 (*n* = 218)	19.6 (16.8–22.5)	13.4 (10.5–16.3)	17.0 (15.0–19.1)	>/=50 (*n* = 253)	22.0 (19.1–25.0)	16.6 (13.4–19.8)	19.8 (17.6–22.0)	>/=50 (*n* = 220)	19.5 (16.6–22.3)	13.9 (11.0–16.9)	17.2 (15.1–19.3)	>/=50 (n = 220)	30.1 (26.8–33.4)	22.6 (19.0–26.1)	27.0 (24.5–29.4)
Total	14.9 (13.7–16.3)	12.9 (11.2–14.6)	14.3 (13.2–15.3)	Total	17.1 (15.7–18.5)	14.4 (12.6–16.1)	16.2 (15.1–17.3)	Total	12.9 (11.8–14.2)	11.2 (9.6–12.8)	12.4 (11.4–13.3)	Total	23.2 (21.6–24.7)	20.1 (18.1–22.1)	22.1 (20.9–23.3)

*CI* = confidence interval; *DIS* = difficulty initiating sleep; *DMS* = difficulty maintaining sleep; *EMA* = early morning awakening

Table 3 shows the factors that are independently associated with each type of insomnia symptoms. Male gender, education level, rural residence, and being unemployed or retired were negatively associated with one or more types of insomnia symptoms. Lacking health insurance, older age and more severe depressive symptoms were positively associated with one or more types of insomnia symptoms.

**Table 3:** Adjusted odds ratios and 95% confidence intervals of sociodemographic characteristics associated with any type of insomnia symptoms

	DIS			DMS			EMA		
	*p*	OR	95%CI	*p*	OR	95%CI	*p*	OR	95%CI
Male gender	**0.04**	**0.78**	**0.61–0.99**	**0.04**	**0.79**	**0.64–0.99**	0.20	0.85	0.67–1.09
Marital status									
Single	–	1.0	–	–	1.0	–	–	1.0	–
Married	0.17	0.80	0.59–1.09	0.92	1.02	0.75–1.37	0.42	1.16	0.81–1.66
Divorced/widowed	0.35	1.27	0.76–2.13	0.15	1.44	0.87–2.38	0.24	1.39	0.80–2.42
Rural residence	**< 0.001**	**0.72**	**0.58–0.89**	**< 0.001**	**0.69**	**0.57–0.85**	**< 0.001**	**0.72**	**0.58–0.90**
Employment status									
Employed	–	1.0	–	–	1.0	–	–	1.0	–
Unemployed	0.62	0.94	0.72–1.21	**0.04**	**0.77**	**0.60–0.99**	0.31	0.87	0.66–1.14
Retired	0.71	0.94	0.66–1.32	**0.03**	**0.70**	**0.51–0.97**	0.37	0.85	0.60–1.21
Living alone	0.61	1.09	0.79–1.51	0.42	1.14	0.83–1.56	0.24	1.24	0.87–1.77
Personal monthly income >/= 6000 (Yuan)	0.62	0.94	0.72–1.22	0.12	0.82	0.64–1.06	0.14	0.81	0.61–1.07
No health insurance	**< 0.001**	**1.43**	**1.15–1.78**	0.52	1.07	0.88–1.30	0.46	1.09	0.87–1.36
Family history of psychiatric disorders	0.09	1.39	0.96–2.01	0.12	1.33	0.92–1.91	0.15	1.33	0.90–1.97
Current drinking	0.07	1.24	0.98–1.58	0.83	1.03	0.81–1.29	0.55	0.92	0.71–1.20
Current smoking	0.13	1.26	0.93–1.71	0.64	1.07	0.80–1.44	0.99	1.0004	0.72–1.40
Age (years)	**< 0.001**	**1.02**	**1.01–1.03**	**< 0.001**	**1.03**	**1.02–1.03**	**< 0.001**	**1.03**	**1.02–1.04**
Education (years)	**< 0.001**	**0.95**	**0.92–0.97**	**< 0.001**	**0.96**	**0.94–0.99**	**< 0.001**	**0.96**	**0.93–0.99**
GAD-7 total	0.29	1.02	0.99–1.04	0.49	1.01	0.98–1.04	0.61	1.01	0.98–1.04
PHQ-9 total	**< 0.001**	**1.19**	**1.15–1.22**	**< 0.001**	**1.19**	**1.16–1.23**	**< 0.001**	**1.18**	**1.15–1.22**

Bolded values: *p* < 0.05; *CI* = confidence interval; *DIS* = difficulty initiating sleep; *DMS* = difficulty maintaining sleep; *EMA* = early morning awakening; *GAD-7* = Generalized Anxiety Disorder Scale-7; *OR* = odds ratio; *PHQ-9* = Patient Health Questionnaire-9

## Discussion

This was the first study that examined the prevalence of insomnia symptoms and their association with demographic correlates in Chinese patients attending medical outpatients clinics attached to general hospitals. The prevalence of any type of insomnia symptoms was 22.1%, which is higher than the figure (15.0%) reported in a meta-analysis of 17 general population studies in China [1]. Medical conditions are recognized independent contributors to insomnia [9, 30]. Neurological, cardiovascular, gastrointestinal, and gynecological diseases result in limited physical or outdoor activity, exposure to bright light, and increased daytime sleep, all of which could lead to night-time insomnia symptoms. The prevalence of insomnia symptoms in this study is significantly higher than the figure (11.7%) in Japanese outpatients [12]. Differences across studies could be partly due to inconsistencies in the definitions of insomnia symptoms, time frame (e.g., current, past month, past year or lifetime prevalence), as well as types of physical diseases and sampling methods. Therefore, comparisons of findings across studies should be made with caution.

Only 17.5% of patients with insomnia symptoms in this study reported to taking sleeping pills, which is higher than Chinese nurses (11.7%) [31] and the Chinese general population (5.4%) [9]. The relatively low treatment rate may be attributed to the lack of access to treatment and the reluctance in seeking help among in Chinese people since they generally do not regard insomnia as a medical condition [9, 32].

An independent association between depressive symptoms and insomnia symptoms were found in this study, which is consistent with earlier findings in both Asian [9, 12, 33] and Western settings [7, 30, 34]. However, the causality between depressive and insomnia symptoms could not been identified due to the cross-sectional study design and the fact that poor sleep is a core feature of depressive disorder [35]. As expected, advanced age and lack of health insurance were positively associated with insomnia symptoms.

This study also confirmed prior findings [36, 37] that people who lived in rural settings had less insomnia symptoms than their urban counterparts. In urban areas noise pollution, leisure activities at night, and shift work are frequently unavoidable [37]; in contrast, rural residents are more likely to have frequent outdoor activities and exposure to natural daylight, which could improve sleep quality [38]. Consistent with the earlier findings in general population [9, 39], this study found that insomnia symptoms were associated with lower education level. Persons with lower education level often face greater work-related stress, which may lead to insomnia symptoms. This study did not confirm previous findings that low income [40] was associated with insomnia symptoms.

The results of this study should be treated with caution due to several methodological shortcomings. First, due to logistical reasons, this study was only conducted in outpatient clinics attached to four general hospitals in Guangzhou, therefore, the findings could not be generalized to all patients in the participating hospitals. Second, due to the cross-sectional design, the causality between insomnia symptoms and other variables could not be explored. Third, the sample was heterogeneous in terms of the primary diagnoses and the severity of medical conditions, which could not be controlled for. Fourth, certain measures, such as the presence and frequency of insomnia symptoms, were self-reported, which could be subject to recall bias. Fifth, reasons for outpatient visits were not recorded. In addition, primary sleep disorders other than insomnia symptoms were not examined. Finally, several variables related to insomnia symptoms, such as the presence and severity of medical conditions and primary sleep disorders, sleep quality and the level of sleep hygiene, frequency of nocturnal micturition, use of psychotropic medications, work schedule (such as shifting work), were not collected or evaluated. The strengths of this study include the large sample size and the standardized method of data collection.

## Conclusion

Insomnia symptoms are common in Chinese patients attending medical outpatient clinics attached to general hospitals. In particular, older age, female gender, urban residency, employment, lower education level, lack of health insurance, and more severe depressive symptoms appear to increase the risk of insomnia symptoms. Regular screening for insomnia symptoms should be a routine part of care in Chinese outpatient clinics. Sleep hygiene, pharmacotherapy and psychosocial interventions for insomnia symptoms should be given more attention in China. Further surveys are warranted to examine the situation in other parts of China. Longitudinal studies are also needed to determine the demographic and clinical predictors of insomnia symptoms.

## Additional file

Additional file 1: The number of patients from the four outpatient clinics. (DOCX 31 kb)

## Abbreviations

CI: Confidence interval
DIS: Difficulty initiating sleep
DMS: Difficulty maintaining sleep
EMA: Early morning awakening
GAD-7: Generalized Anxiety Disorder 7 items
PHQ-9: Patient Health Questionnaire 9
QOL: Quality of life
