# Supplementary material for: Prevalence of insomnia symptoms and their associated factors in patients treated in outpatient clinics of four general hospitals in Guangzhou, China
Source: BMC Psychiatry. 2018 Jul 18;18:232. doi: 10.1186/s12888-018-1808-6 (PMC6052511; doi:10.1186/s12888-018-1808-6)
Supplement: Supplementary file 1 — The number of patients from the four outpatient clinics. (DOCX 31 kb) [file 12888_2018_1808_MOESM1_ESM.docx]

**Additional file 1.** The number of patients from the four outpatient clinics ^a^

|  | Total sample  (n=4339; %) | A (%) | B (%) | C (%) | D (%) |
| --- | --- | --- | --- | --- | --- |
| Neurological outpatient clinic | 1183 (26.9) | 91 (100) | 330 (25.6) | 376 (24.9) | 386 (325.5) |
| Gastrointestinal outpatient clinic | 1057 (24.0) | 0 (0) | 302 (23.4) | 379 (25.1) | 376 (24.9) |
| Cardiovascular outpatient clinic | 1047 (23.8) | 0 (0) | 324 (25.2) | 350 (23.2) | 373 (24.7) |
| Gynaecological outpatient clinic | 1112 (25.3) | 0 (0) | 332 (25.8) | 403 (26.7) | 377 (24.9) |
| Male gender | 1503 (34.2) | 32 (35.2) | 395 (30.7) | 551 (36.5) | 525 (34.7) |
| Age (years) | 41.8±15.9 | 33.4±14.0 | 39.6±14.8 | 40.3±14.7 | 45.6±17.3 |
| Education (years) | 10.3±4.2 | 13.4±4.0 | 11.1±3.9 | 10.3±4.2 | 9.3±4.4 |
| Any insomnia symptoms | 973 (22.1) | 24 (26.4) | 327 (25.4) | 334 (22.1) | 288 (19.0) |
| DIS | 628 (14.3) | 19 (20.9) | 211 (16.4) | 232 (15.4) | 166 (11.0) |
| DMS | 711 (16.2) | 19 (20.9) | 240 (18.6) | 239 (15.8) | 213 (14.1) |
| EMA | 544 (12.4) | 14 (15.4) | 180 (14.0) | 175 (11.6) | 175 (11.6) |
| Abbreviation: A=the Third Affiliated Hospital of Sun Yet-Sen University; B=the First Affiliated Hospital of Guangzhou University of Traditional Chinese Medicine; C=Nanfang Hospital (Southern Medical University); D=Guangzhou Panyu Central Hospital, DIS=difficulty initiating sleep; DMS=difficulty maintaining sleep; EMA=early morning awakening.  ^a^Three hospitals (B, C, and D) recruited participants from the gastrointestinal, cardiovascular, and gyanecological outpatient clinics; and all four hospitals recruited from their neurological outpatient clinics. | | | | | |
